# Minimalist ensemble algorithms for genome-wide protein localization prediction

**DOI:** 10.1186/1471-2105-13-157

**Published:** 2012-07-03

**Authors:** Jhih-Rong Lin, Ananda Mohan Mondal, Rong Liu, Jianjun Hu

**Affiliations:** 1 Department of Computer Science and Engineering, University of South Carolina, Columbia, SC, 29208, USA; 2 Department of Mathematics and Computer Science, Claflin University, Columbia, SC, 29115, USA

**Keywords:** Protein subcellular localization, Ensemble algorithms, Classifiers, Logistic regression

## Abstract

**Background:**

Computational prediction of protein subcellular localization can greatly help to elucidate its functions. Despite the existence of dozens of protein localization prediction algorithms, the prediction accuracy and coverage are still low. Several ensemble algorithms have been proposed to improve the prediction performance, which usually include as many as 10 or more individual localization algorithms. However, their performance is still limited by the running complexity and redundancy among individual prediction algorithms.

****Results**:**

This paper proposed a novel method for rational design of minimalist ensemble algorithms for practical genome-wide protein subcellular localization prediction. The algorithm is based on combining a feature selection based filter and a logistic regression classifier. Using a novel concept of contribution scores, we analyzed issues of algorithm redundancy, consensus mistakes, and algorithm complementarity in designing ensemble algorithms. We applied the proposed minimalist logistic regression (LR) ensemble algorithm to two genome-wide datasets of Yeast and Human and compared its performance with current ensemble algorithms. Experimental results showed that the minimalist ensemble algorithm can achieve high prediction accuracy with only 1/3 to 1/2 of individual predictors of current ensemble algorithms, which greatly reduces computational complexity and running time. It was found that the high performance ensemble algorithms are usually composed of the predictors that together cover most of available features. Compared to the best individual predictor, our ensemble algorithm improved the prediction accuracy from AUC score of 0.558 to 0.707 for the Yeast dataset and from 0.628 to 0.646 for the Human dataset. Compared with popular weighted voting based ensemble algorithms, our classifier-based ensemble algorithms achieved much better performance without suffering from inclusion of too many individual predictors.

**Conclusions:**

We proposed a method for rational design of minimalist ensemble algorithms using feature selection and classifiers. The proposed minimalist ensemble algorithm based on logistic regression can achieve equal or better prediction performance while using only half or one-third of individual predictors compared to other ensemble algorithms. The results also suggested that meta-predictors that take advantage of a variety of features by combining individual predictors tend to achieve the best performance. The LR ensemble server and related benchmark datasets are available at http://mleg.cse.sc.edu/LRensemble/cgi-bin/predict.cgi.

## Background

Functions of proteins are closely correlated with their subcellular locations. For example, Assfalg et al. [1] showed that there exists strong correlation between localization and proteins fold and localization can be utilized to predict structure class of proteins. It is thus desirable to accurately annotate subcellular location of proteins to elucidate their functions. In the past ten years, dozens of protein localization algorithms have been proposed based on different information sources such as amino acid composition, sorting signals, functional motifs, conserved domains, homology search, and protein-protein interaction [2]. A variety of machine learning techniques, such as SVM and K-nearest neighbour classifiers, have been used in these prediction algorithms. Although existent methods have achieved success at different degrees, a comprehensive evaluation study has shown that many of the reported prediction accuracies are far from being sufficient for genome wide protein localization prediction [3].

Recently, several research groups proposed to apply ensemble or integration of algorithms to protein localization prediction [4-8]. Liu et al. [4] proposed weighted and adaptive weighted voting algorithms in which the overall accuracy of a standalone algorithm is used as the weight. Laurila and Vihinen [5] proposed an integrated method (PROlocalizer) which combines the predictions of multiple specialized binary localization prediction algorithms such as TMHMM and Phobius. Park et al. [6] developed a Linear Discriminant Analysis (LDA) method (ConLoc) to assign LDA optimal weights for weighted voting. Assfalg et al. [7] proposed two ensemble localization algorithms; one is a scored voting scheme based on the ranks of the prediction accuracy of the predictors; the other chose J48 decision tree (DT) classifier as the integration scheme. Shen and Burger [8] proposed a two-layer decision tree method to improve the prediction accuracy of a single subcellular location. Most of these ensemble algorithms integrated 10 or more standalone prediction methods for localization prediction without considering their relationships such as redundancy and complementarity. This makes these ensemble algorithms computationally intensive. Furthermore, incorporation of unnecessary predictors into an ensemble algorithm may overfit the training data and result in degradation of its prediction performance, which has been reported recently for ensemble mitochondrion predictors [9].

In this paper, we evaluated 9 standalone localization prediction algorithms and analyzed their bias and relationships in the prediction space of the resulting ensemble algorithms. We found that ensemble algorithms based on the combination of several specific predictors achieved comparable prediction performance as using all 9 predictors, suggesting that a high degree of redundancy exists among all individual predictors. We thus proposed a minimalist ensemble prediction algorithm for subcellular localization prediction and evaluated its performance on two data sets, which showed high performance and significant reduction of computational complexity and running time.

## Methods

### Standalone protein localization predictors

To implement our ensemble localization predictor, we selected 8 published localization prediction algorithms provided that the software or web server is publicly available, and batch submission is supported. These algorithms include YLoc [10], MultiLoc2 [11], KnowPred [12], Subcell [13], WoLFPSORT [14], BaCelLo [15], CELLO [16], SubLoc [17]. We also included NetLoc [18], a protein-protein interaction (PPI) based prediction method. These prediction methods differ in the features that characterize proteins targeting different subcellular locations (Table 1) and the prediction algorithms. These diverse features include sorting signals, amino acid composition, known motifs or domains, homology search against a known dataset or database such as SwissProt, evolutionary information such as phylogenetic profiles or sequence profiles, and protein-protein interaction. The overlap of the used features among localization predictors suggests that redundant predictions could be made when these prediction methods are combined to build an ensemble algorithm, which could mislead the prediction behaviour of the resulting ensemble algorithm.

**Table 1:** Features used in localization prediction algorithms

	**Sorting signal**	**Amino acid composition**	**Known domains or motifs**	**Homology search**	**Evolutionary information**	**PPI**
NetLoc						X
YLoc	X	X	X	X		
MultiLoc2	X	X	X		X	
KnowPred				X		
Subcell		X				
WoLFPSORT	X	X	X			
BaCelLo		X			X	
CELLO		X				
SubLoc		X				

In addition to amino acid sequence information, protein-protein interaction has been known as external information correlated to protein subcellular localization. A number of algorithms have been developed to utilize PPI features to predict protein localization (Hishigaki et al. [19], Lee et al. [20] and Shin et al. [21]). Recently, our group developed NetLoc [18], a kernel-based logistic regression (KLR) method, which can effectively extract PPI features to predict protein localization. Considering that NetLoc simply used PPI as its features, we integrated NetLoc into our ensemble algorithms to compare the ensemble performances with and without a PPI-based predictor. In our experiments, PPI data of NetLoc is based on the whole Saccharomyces cerevisiae physical PPI dataset obtained from BioGRID database [22]. We exclude proteins overlapped with our Yeast datasets from the PPI dataset to ensure independency between the training and testing datasets.

### Mapping of subcellular locations

Different localization predictors may have different subcell resolutions. In order to compare their performances on genome wide datasets, we applied a location mapping scheme to map the subcellular locations of standalone predictors to unified 5 locations in the ensemble algorithms, including Cytosol, Mitochondrion, Nucleus, Secretory (secretory pathway), and *Others*. Six classes of subcellular locations are mapped to Secretory according to [11]: extracellular, plasma membrane, endoplasmic reticulum, golgi apparatus, lysosomal, and vacuolar. Except for Cytosol, Mitochondrion, Nucleus, and Secretory, the remaining subcellular locations are categorized as *Others*. For example, for CELLO, the following subcellular locations are mapped to Secretory: extra, plas, er, vacu, golgi, and lyso; chlo, pero, and cytos are mapped to *Others*. For WoLFPSORT, E.R., extr, plas, golg, lyso, and vacu are mapped to Secretory; chlo, cysk, and pero are mapped to *Others*.

### Contribution score

To explore the complementary relationship among the individual predictors used in an ensemble algorithm, we calculated contribution scores [23] of component standalone prediction methods. This measure is used to evaluate the contribution of each individual classifier to the ensemble algorithm, and has been used for pruning large ensemble set. The main idea of the contribution score is that predictors that tend to make correct and minority predictions among other predictors will be scored higher since they make unique contribution and thus are essential for the ensemble algorithm. On the other hand, predictors with low contribution scores tend to make incorrect and majority predictions. The contribution score of a predictor in an ensemble algorithm is calculated as follows:

(1) $$\text{Contribution score of predictor i}=\sum_{j=1}^{N}\left(\alpha_{\mathrm{ij}}\left(2\upsilon_{\max}^{\left(j\right)}-\upsilon_{p_{i}\left(protein_{j}\right)}^{\left(j\right)}\right)+\beta_{\mathrm{ij}}\upsilon_{\sec}^{\left(j\right)}+\theta_{\mathrm{ij}}(\upsilon_{\text{correct}}^{\left(j\right)}-\upsilon_{p_{i}\left(protein_{j}\right)}^{\left(j\right)}-\upsilon_{\max}^{\left(j\right)}\right)$$

where:

(2) $$\begin{array}{l} \alpha_{\mathrm{ij}}=\left\{ \begin{array}{l} 1\;\text{if}\;p_{i}\left(protein_{j}\right)=real_{j}\;\text{and}\;p_{i}\left(protein_{j}\right)\;\text{is in}\;\text{the minority}\;\text{group;}\\ 0\text{otherwise}\text{. } \end{array}\right.\\ \beta_{\mathrm{ij}}=\left\{ \begin{array}{l} 1\;\text{if}\;p_{i}\left(protein_{j}\right)=real_{j}\;\text{and}\;p_{i}\left(protein_{j}\right)\;\text{is in}\;\text{the majority}\;\text{group;}\\ \text{ 0 otherwise}\text{. } \end{array}\right.\\ \theta_{\mathrm{ij}}=\left\{ \begin{array}{l} 1\;\text{if}\;p_{i}\left(protein_{j}\right)\neq real_{j};\\ \text{ 0 otherwise}\text{. } \end{array}\right. \end{array}$$

Symbols in the formula are explained as follows: for a protein j, the prediction results of nine predictors in the order of predictor 1 to predictor 9 are Cytosol, Nucleus, Nucleus, Mitochondrion, Nucleus, Cytosol, Nucleus, Nucleus, and Nucleus, while the real localization of protein j is Cytosol. In this case, the majority votes (predictions) are for Nucleus, the number of the majority votes is denoted as $\upsilon_{\max}^{\left(j\right)}$, which is 6; the number of the second majority votes is denoted as $\upsilon_{\sec}^{\left(j\right)}$, which is 2; the number of the correct votes is denoted as $\upsilon_{\text{correct}}^{\left(j\right)}$, which is 2; the prediction result of predictor i is denoted as $p_{i}\left(protein_{j}\right)$; the number of predictors having the same prediction result with predictor i is denoted as $\upsilon_{p_{i}\left(protein_{j}\right)}^{\left(j\right)}$. From the formula, we can see that predictor 1 and predictor 6 have the same positive contribution, which is 2*6-2 = 10; predictor 4 has minor negative contribution, which is −5; predictors 2,3,5,7,8,9 have the most negative contribution, which is −10. If the dataset used to learn contribution scores has N proteins, then the final contribution score of a predictor is summation of its N contributions. We normalized the final contribution scores (CS) with the formula: (CS – μ)/σ, where μ and σ are mean and standard deviation of contribution scores among predictors.

### Minimalist ensemble prediction algorithm

Existing ensemble algorithms tend to include as many as possible component classifiers for better prediction performance. However, including redundant predictors not only increases computational complexity and collecting effort, but also may lead to over-fitting [9]. Moreover, predictors with poor performance could mislead the ensemble algorithms especially those using majority voting schemes. It is thus desirable to find the minimal subset of predictors for achieving equally good or better prediction performance.

Several strategies can be used to find the minimal set of predictors: exhaustive search of all possible combinations of component predictors, feature selection, and selecting top k most accurate predictors. We did an exhaustive search for all combinations of K individual predictors to build different ensemble algorithms. It shows that combining 6 out of 9 predictors can achieve the best performance when the logistic regression classifier was used to integrate the predictions. However, exhaustive search is a time consuming process especially when the set of available predictors is large. Top-K accuracy selection method is straightforward and fast, but has the limitation of neglecting the redundancy among individual predictors.

Here we proposed a minimalist ensemble design method to approximate the smallest set of predictors with the best possible prediction accuracy. The rationale is to find the smallest subset of predictors whose predictions are highly correlated to the real locations. The minimalist ensemble design problem is similar to feature selection when the prediction labels of individual predictors are considered as features. Here, we chose the correlation based feature subset evaluator (CfsSubsetEval) [24] as the attribute evaluator to evaluate correlation between a feature subset and the class. Greedy-Stepwise method is used to search optimal feature subsets in different size of K through the space: the starting point of search is set as the set with all available predictors (assume size N). Each time Greedy-Stepwise algorithm will remove one feature or predictor from the set which would produce a reduced set with the highest possible CfsSubsetEval Score. We continue the process until set size is 1, while along the way the predictors in the set with size K are recorded as the output of our minimalist ensemble algorithm. After the K individual predictors are selected based on the training dataset, their predicted localizations for all proteins in the training dataset will be used as features, and a machine learning based classifier, such as naive Bayes, logistic regression, or decision trees is used to train a classifier to predict the final subcellular localization. This method used to select minimalist set of individual predictors can also be used for building ensemble algorithms based on weighted voting or LDA.

### Datasets preparation

Two genome-wide protein localization databases are used to build three datasets in our experiments. The yeast dataset is obtained from Huh et al. [25]. We excluded proteins localized to *Others* (after location mapping) and multi-location proteins from the yeast dataset. Two versions of the yeast dataset with different resolutions are prepared; for the low-resolution yeast dataset (Yeast Low-Res), we extracted proteins in Cytosol, Nucleus, Mitochondrion, Secretory after location mapping. For the high-resolution yeast dataset (Yeast High-Res), we extracted proteins in Cytosol, Nucleus, Mitochondrion, ER, Vacuole, Golgi, and Cell Periphery (plasma membrane and extracellular). The Human dataset is obtained from the LOCATE database [26] by extracting proteins in 4 locations (Cytoplasmic, Mitochodria, Nuclear, and Extracellular). Then we removed all multi-location proteins. For both Yeast and Human datasets, Blastclust with 30% sequence identity was used to remove redundant sequences. In addition, proteins overlapped with the training datasets of component predictors in the corresponding ensemble experiment are removed. It should be noted that the Yeast High-Res dataset is highly overlapped with the Yeast Low-Res datasets. The final distribution of proteins in different locations for the three datasets is shown in Table 2.

**Table 2:** The distributions of proteins in different locations for the test datasets

**Dataset**	**Cytosol**	**Mitochondrion**	**Nucleus**	**Secretory**				**Total**
Yeast-LowRes	498	175	234	315				1222
Human	361	327	159	458				1305
	**Cytosol**	**Mitochondrion**	**Nucleus**	**ER**	**Vacuole**	**Golgi**	**Cell Periphery**	
Yeast-HighRes	530	165	233	149	103	33	34	1247
^1^Overlap	451	133	218	132	90	32	0	1056

^1^Overlap of Yeast LowRes and Yeast HighRres.

### Evaluation of individual predictors and ensemble algorithms

To evaluate the performance of predictors, accuracy and MCC were calculated using the equations below:

(3) $$\text{Accuracy}:\frac{\left(TP+TN\right)}{\left(TP+TN+FP+FN\right)}$$

(4) $$\text{MCC}:\frac{\left(TP\times TN-FP\times FN\right)}{\sqrt{\left(TP+FN\right)\left(TP+FP\right)\left(TN+FP\right)\left(TN+FN\right)}}$$

where TP, TN, FP, FN means true positive, true negative, false positive and false negative predictions. It should be noted that since localization prediction is a multi-class classification problem, MCC can only be calculated for each location while an overall accuracy can be calculated for each prediction method for a given dataset. In our experiments, 10-fold cross-validation was used to evaluate all the ensemble algorithms.

## Results and discussion

### Evaluation of individual predictors

We obtained the prediction results on three test datasets (Yeast Low-Res, Yeast High-Res and Human) from the selected individual predictors using the web servers or standalone programs and then evaluated their accuracy and MCC scores. The results of 9 predictors for the Yeast Low-Res dataset are shown in Table 3, the results of 6 predictors for the Yeast High-Res dataset are shown in Table 4, and the results of 8 predictors for the Human dataset are shown in Table 5.

**Table 3:** Prediction performance (MCC Scores) of individual predictors for the Yeast Low-Res dataset

	**YLoc (2010)**	**NetLoc (2010)**	**MultiLoc2 (2009)**	**KnowPred (2009)**	**Subcell (2008)**	**WoLFPSORT (2007)**	**BaCelLo (2006)**	**CELLO (2006)**	**SubLoc (2001)**	**LR with 8 predictors without NetLoc**	**LR with all 9 predictors**
Cytosol	0.146	0.270	0.268	0.286	0.134	0.265	0.164	0.261	0.184	0.429	0.504
Mitochondrion	0.556	0.350	0.581	0.415	0.243	0.549	0.526	0.547	0.354	0.668	0.666
Nucleus	0.367	0.484	0.420	0.345	0.181	0.312	0.291	0.302	0.260	0.476	0.550
Secretory	0.314	0.473	0.339	0.534	0.326	0.568	0.339	0.534	0.391	0.607	0.664
Overall Accuracy	0.453	0.556	0.558	0.51	0.399	0.484	0.468	0.493	0.439	0.668	0.707

**Table 4:** Prediction performance (MCC Scores) of individual predictors for the Yeast High-Res dataset

	**YLoc (2010)**	**MultiLoc2 (2009)**	**Subcell (2008)**	**WoLFPSORT (2007)**	**CELLO (2006)**	**NetLoc (2010)**	**LR with 5 predictors without NetLoc**	**LR with all 6 predictors**
Cytosol	0.441	0.293	0.146	0.251	0.255	0.247	0.459	0.555
Mitochondrion	0.689	0.496	0.251	0.510	0.501	0.318	0.684	0.713
Nucleus	0.405	0.275	0.181	0.311	0.306	0.434	0.351	0.473
ER	0.207	0.203	0.022	0.059	0.000	0.340	0.431	0.463
Vacuole	0.115	0.045	0.034	0.000	0.061	0.189	0.174	0.191
Golgi	0.008	0.010	0.054	0.118	−0.005	0.465	0.038	0.275
Cell Periphery	0.107	0.044	0.068	0.142	0.090	0.449	0.04	0.269
Overall accuracy	0.506	0.473	0.300	0.362	0.354	0.523	0.585	0.640

**Table 5:** Prediction performance (MCC Scores) of individual predictors for the Human dataset

	**YLoc (2010)**	**MultiLoc2 (2009)**	**KnowPred (2009)**	**Subcell (2008)**	**WoLFPSORT (2007)**	**BaCelLo (2006)**	**CELLO (2006)**	**SubLoc (2001)**	**LR with all 8 predictors**
Cytosol	0.308	0.334	0.307	0.050	0.261	0.220	0.117	0.065	0.362
Mitochondrion	0.546	0.451	0.048	0.080	0.329	0.439	0.369	0.264	0.515
Nucleus	0.454	0.293	0.419	0.122	0.277	0.233	0.234	0.162	0.375
Secretory	0.720	0.627	0.477	0.205	0.553	0.607	0.428	0.339	0.712
Overall Accuracy	0.628	0.581	0.514	0.303	0.527	0.54	0.419	0.375	0.646

For the Yeast dataset (Tables 3, 4), most algorithms have better performance on predicting Mitochondrion proteins. For the Yeast High-Res dataset (Table 4), we can see that all predictors except NetLoc showed poor performance on predicting proteins localized to secretory pathway compartments especially golgi, and cell periphery. This suggests that PPI can be an effective feature for predicting low-resolution compartments. Predictors with relatively high accuracy on the Yeast Low-Res Secretory proteins, such as CELLO and WoLFPSORT, don’t have corresponding performance on predicting proteins localized to ER, Golgi, Vacuole in the Yeast High-Res dataset which are highly overlapped with the Yeast Low-Res Secretory proteins (Table 3). This means those predictors have difficulties in distinguishing smaller compartments of secretory pathway. YLoc and MultiLoc2 have very different performances between the Yeast Low-Res and High-Res datasets, which could be due to the use of different training datasets. For the Human dataset (Table 5), the Secretory proteins (which are exclusively Extracellular proteins) are the easiest for YLoc, MultiLoc2, and WoLFPSORT, which may suggest that these proteins have more distinct features such as secretory pathway signals than the Yeast Secretory proteins. As shown in Table 1, YLoc, MultiLoc2, and WoLFPSORT all use sorting signals as one of their features. The variation of prediction performance of the individual predictors implies that an ensemble algorithm may be able to integrate their strengths and achieve better overall performance.

### Ensemble performance

From Tables 3, 4, 5 we can compare the performances between logistic regression (LR) ensemble algorithms and their element predictors on the three test datasets. We can see that LR ensemble has better overall accuracy than the best element predictor over the three datasets; for the Yeast Low-Res dataset and Yeast High-Res dataset, LR ensemble have more than 10% improvement over the best element predictors when integrating all available element predictors. However, LR ensemble does not always have the best performance on each compartment. This is because the ensemble training process is to optimize the overall accuracy while performance of certain compartment(s) could be compromised. We can also see that when all of the element predictors failed on certain compartments, such as Golgi and Cell Periphery in the Yeast High-Res dataset, LR ensemble doesn’t have any improvement on predicting those compartments.

### Prediction performance of the optimal ensemble algorithms using exhaustive search

**Figure 1:**
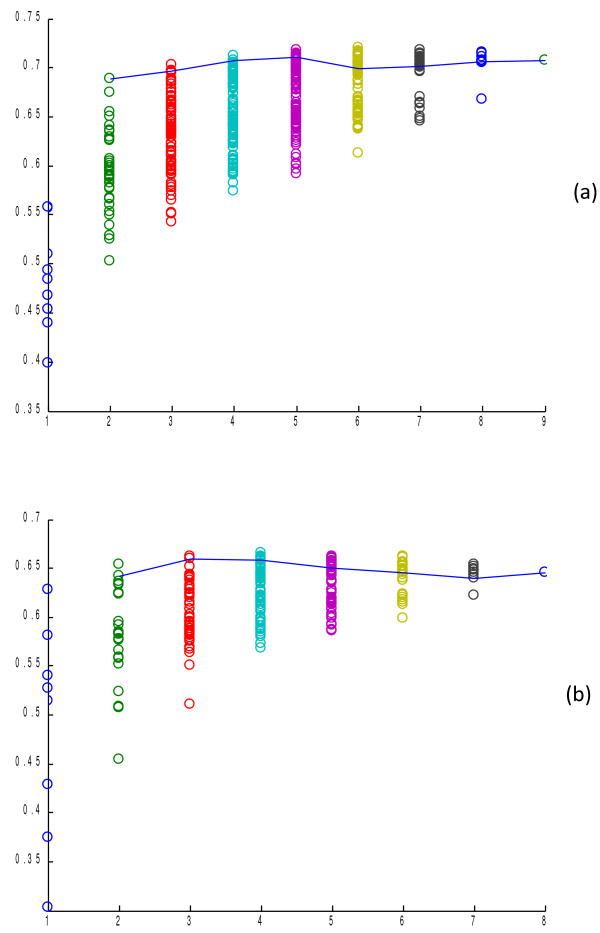
**Prediction performance of the logistic regression ensemble methods with K individual predictors selected by exhaustive search.** (**a**) Performance on the Yeast Low-Res dataset, (**b**) Performance on the Human dataset. Each dot represents one combination of predictors. The number of predictors is annotated on the X axis. The performance of the logistic regression ensemble method is annotated on the Y axis. The dots connected by the line represent the combinations of predictors determined by the minimalist algorithm for different K values.

Here we evaluated the prediction accuracy of the logistic regression ensemble algorithm with all combinations of K (K = 2…9) predictors using 10-fold cross-validation. Figure 1 (a) shows the result tested on the Yeast Low-Res dataset. First, we found that by using just three predictors, the ensemble algorithm can achieve comparable performance as using nine predictors. The 3 predictors are NetLoc (PPI), WoLFPSORT and YLoc which cover most of the available features among the predictors. On the other hand, the ensemble algorithm composed of predictors with low coverage of features has poor prediction efficiency. It is also observed that when more predictors were used, the performance discrepancy between the ensemble algorithms based on different predictors became smaller. This indicates that the prediction performance is more reliable as the number of predictors increases.

We also evaluated the ensemble performance on the Human dataset with all combinations of predictors including YLoc, MultiLoc2, WoLFPSORT, CELLO, SubLoc, Subcell, BaCelLo and KnowPred. However, relatively limited accuracy improvement over the best individual predictor has been achieved by the LR ensemble compared to the Yeast dataset. One reason is that the ensemble algorithm for the Yeast dataset includes NetLoc which uses protein-protein correlation network information for localization prediction. This distinctive feature makes it complementary to the other algorithms, which leads to significant performance boosting. Another reason may be that the strengths and bias of different predictors are enlarged or reduced to different degrees on different datasets, which may result in the change of complementary relationship among predictors. The varying complementary relationship thus leads to different prediction accuracy of the ensemble composed of the same set of predictors on different datasets.

### Contributions of individual predictors to the ensemble algorithm

**Figure 2:**
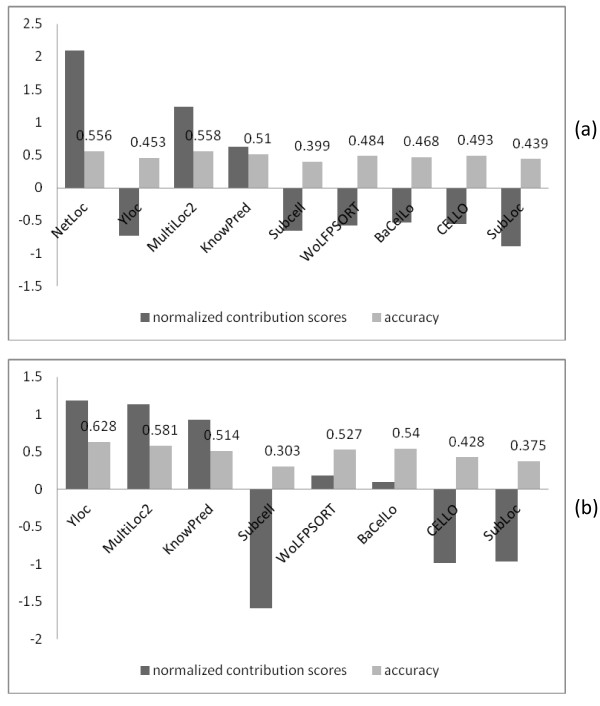
**Contribution scores of individual predictors.** (**a**) 9 predictors for the Yeast Low-Res dataset, (**b**) 8 predictors for the Human dataset.

To explore the contributions of individual predictors to the ensemble algorithm and their redundant or complementary relationships, we calculated their contribution scores in the ensemble algorithm for the Yeast Low-Res and Human datasets. Nine predictors are available for the Yeast Low-Res dataset and 8 predictors for the Human dataset. Figure 2(a) and (b) show the normalized contribution scores and prediction accuracies of the 9 (8) predictors on the Yeast Low-Res dataset and Human dataset respectively. For the Yeast Low-Res dataset, YLoc, Subcell, WolfPSORT, BaCelLo, CELLO, and SubLoc all have relatively low contribution scores, which suggests that their predictions are highly redundant with the other predictors’ predictions. We also found that the predictors simply using the most common features(amino acids composition) such as CELLO, SubLoc, Subcell, all have relatively low contribution scores, which suggests that the proteins whose localizations can be correctly predicted by these predictors can also be predicted correctly by other predictors. On the other hand, it can be observed that predictors using distinct features such as NetLoc and KnownP have relatively high contribution scores. NetLoc (PPI) has the highest contribution score because it used very different PPI information compared to other predictors, which allows it to correctly predict proteins that other individual predictors cannot. KnowPred applies a sophisticated local similarity method to detect remote sequence homology and therefore might correctly predict some proteins that most of others cannot. Another reason why NetLoc and KnowPred have relatively high contribution scores is that they don’t use other common features so they are less likely to make the same wrong predictions like other predictors. For the Human dataset, YLoc, MultiLoc2 and KnowPred have the highest contribution scores while CELLO, SubLoc, and Subcell still have the lowest contribution scores, which suggests that the latter three predictors’ correct predictions can be covered by the other component predictors or that they tend to mislead the ensemble algorithm by making majority incorrect predictions. This contribution score analysis can thus be applied to evalute future new protein localization predictors in terms of their unique prediction capability.

### Prediction performance of the minimalist ensemble algorithm

To test the performance of our minimalist LR ensemble algorithm with K component predictors, we run the minimalist algorithm to generate the combination of predictors for each K to build the minimalist ensemble algorithms and then tested them on the Yeast Low-Res and Human datasets. The results in Figure 1 show that for the LR ensemble method, our minimalist ensemble algorithm can achieve near-optimal performance for any given K value. We also found that using 3–4 individual predictors can obtain near-best performance for all possible K values on the Yeast Low-Res dataset. This means that our minimalist ensemble algorithm can use 1/2 to 1/3 of individual predictors used by existing ensemble algorithms to achieve similar performance while remarkably reducing the computational effort.

To examine the complementary relationships of the selected algorithms in the ensemble algorithms, Table 6 shows the most frequent predictors selected by the minimalist ensemble algorithms during the 10-fold cross-validation and the best combination for each K according to the exhaustive search of the LR ensemble on the Yeast Low-Res dataset. It is interesting to find that NetLoc and WoLFPSORT are the key component algorithms that are selected by the best combination and the minimalist ensemble with different K components. YLoc is the second tier of algorithms selected by the best combination, while MultiLoc2 is the second tier of algorithm selected by the minimalist algorithm. The consistent difference of the selected component predictors between the best combination and the minimalist after the key component algorithms is due to that our minimalist algorithm used greedy and stepwise method to search the optimal K component predictors.

**Table 6:** The most frequent predictors selected by the minimalist algorithm with size of each K (noted by M) during the 10-fold cross-validation and the best combination of K predictors (noted by B) according to the exhaustive search result of the logistic regression ensemble on the Yeast dataset

**Number of predictors**	**YLoc (2010)**	**NetLoc (2010)**	**MultiLoc2 (2009)**	**KnowPred (2009)**	**Subcell (2008)**	**WoLFPSORT (2007)**	**BaCelLo (2006)**	**CELLO (2006)**	**SubLoc (2001)**
2		BM				BM			
3	B	BM	M			BM			
4	B	BM	BM	M		BM			
5	B	BM	M	BM		BM		M	B
6	BM	BM	M	BM		BM		BM	B
7	BM	BM	M	M	B	BM	BM	BM	B
8	BM	BM	BM	BM	B	BM	M	BM	BM

### Comparison of computational complexity

The computational complexity of the ensemble involves the effort to collect prediction results from individual predictors either from local software running or from web servers and the total running time. Since most of the predictors are available only via web servers which are sometimes offline, it is desirable to have fewer component predictors. As demonstrated in Figure 1, the minimalist algorithm can efficiently find the key component predictors. Since only 4 predictors are needed for the ensemble algorithm to achieve comparable performance of using 9 predictors, about 1/2 to 2/3 amount of computation time to collect prediction results can be saved.

### Comparison of different ensemble schemes

**Figure 3:**
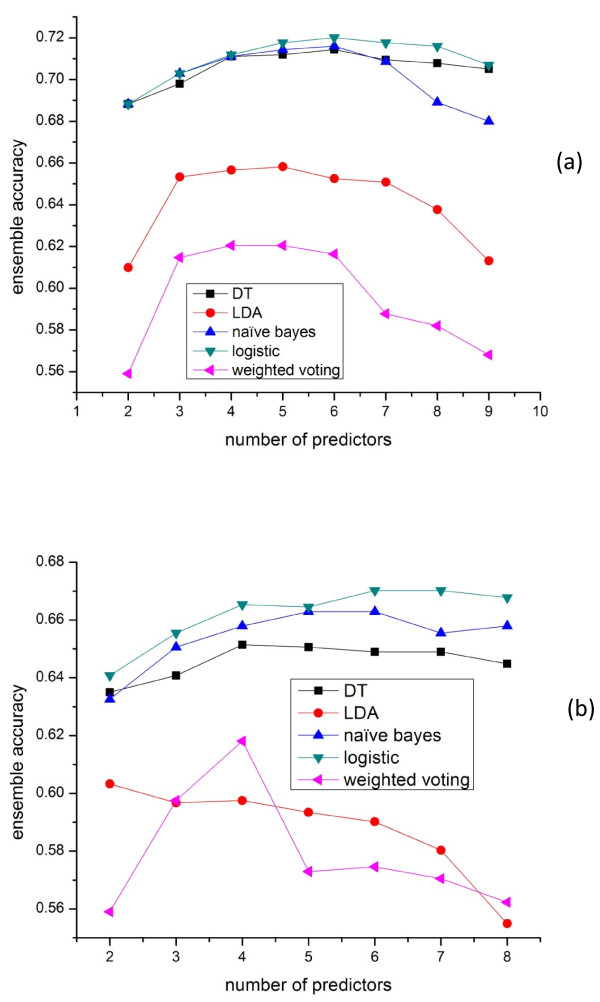
**Performance of the best ensemble on the Yeast dataset using different ensemble schemes with K (K = 2..9) predictors selected by exhaustive search.** (**a**) 9 predictors including NetLoc (PPI) (**b**) 8 predictors without NetLoc (PPI).

Several ensemble schemes have been proposed for building ensemble localization prediction algorithms, including weighted voting [4] (weight is assigned based on predictor accuracy), LDA [6], and classifiers-based ensemble algorithms such as decision tree (DT) [7]. It is interesting to compare their performance on the genome-wide Yeast and Human datasets. Here we compared their best performance given K individual predictors selected by exhaustive search. As shown in Figure 3, weighted voting has the worst performance and its performance degrades dramatically when more individual predictors are included. This is because its prediction can be easily biased by redundant low-performance predictors. LDA ensemble is better than weighted voting because it can assign LDA optimal weights to predictors and avoid the prediction results being biased by low-performance predictors. However, it is still a voting based algorithm which might not be able to capture the rules relating the predictions of predictors to the real locations. For other classifiers-based (such as naive Bayes, decision tree and logistic regression) ensemble methods, they yield better prediction accuracy because these machine learning algorithms can better find and learn the rules between the features (predictions of individual predictors) using supervised learning. For these machine learning ensemble methods, the capability to handle redundancy is essentially the capability to handle over-fitting. As Figure 3 shows, if too many predictors are included, voting based ensemble algorithms such as weighted voting and LDA show the trend of downgrading the performance.

Figure 3(a) and (b) showed the performance of the ensemble algorithms with or without including the PPI based predictor NetLoc. It is observed that ensemble algorithms without NetLoc have much less improvement over the best individual predictors, which means that these ensemble algorithms except weighted voting can automatically take advantage of the unique/beneficial component predictors (such as NetLoc which uses a unique protein-protein interaction features) to improve the performance. From Figure 3(b) we also noticed that LDA ensemble’s performance could degrade dramatically when too many redundant predictors are included without including predictor(s) with distinct property such as NetLoc.

**Figure 4:**
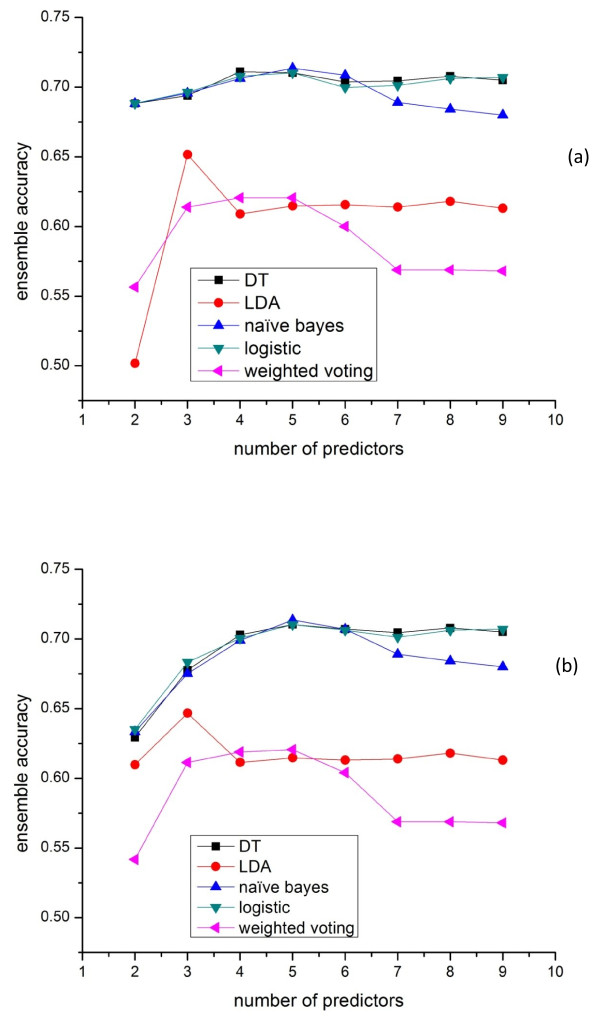
**Performance of different ensemble schemes on the Yeast Low-Res dataset with K (k = 2..9) predictors selected by Minimalist algorithm and Top-K accurate method.** (**a**) Different ensemble methods with K (k = 2..9) predictors selected by Minimalist algorithm. (**b**) Different ensemble methods with K (k = 2..9) predictors selected by Top-K accurate algorithm.

We also compared the performances of the minimalist ensemble algorithms on the Yeast Low-Res dataset. The result is shown in Figure 4(a), which demonstrates similar relationship of the performance for the evaluated ensemble algorithms in Figure 3(a). Figure 4(b) shows the performance of the ensemble methods by selecting the top K accurate predictors. We can see that the main peformance difference between the minimalist ensemble and top-K ensemble is when K is less than 4, which means the top 4 accurate predictors can form a very complimentary group. However, top K method is not reliable especially when the predictor with distinct features has relatively low accuracy, or when many included predictors are highly redundant.

### Comparison with other ensemble algorithms

There are several published and publicly available ensemble algorithms such as ConLoc [6] and PROlocalizer [5]. ConLoc intergrated 13 different predictors and used LDA as the ensemble scheme. PROlocalizer intergrated 11 different programs to predict localization of animal proteins. We tested ConLoc on our Yeast Low-Res and Human datasets. The results are shown in Tables 7 and 8. It should be noted that although our datasets are not overlapped with ConLoc ensemble training dataset, the performance result of ConLoc can still be overestimated since we didn’t exclude proteins of our datasets that are overlapped with the training datasets of ConLoc’s 13 element predictors. To test our minimalist ensemble algorithm, we first collected predictions of ConLoc’s 13 element predictors on the Yeast Low-Res and Human datasets and then tested LR ensemble with 10-fold cross-validation. The results (Tables 7 and 8) showed that LR ensemble achieved higher accuracy than LDA based ConLoc on both datasets, which is consistent with our previous experiment results (Figure 3(a) and 3(b)) alghough ConLoc LDA used a different ensemble training dataset.

**Table 7:** Comparison of the performance of ConLoc and Minimalist LR ensemble algorithm with 13 predictors on the Yeast Low-Res dataset

	**The best element predictor of ConLoc: SherLoc**	**ConLoc**	**LR ensemble with 13 predictors as used in ConLoc**	**LR + minimalist algorithm to select K out of 13 predictors in ConLoc, K = 4**
Cytosol	0.301	0.441	0.489	0.472
Mitochondrion	0.574	0.622	0.708	0.731
Nucleus	0.341	0.461	0.537	0.541
Secretory	0.533	0.537	0.608	0.605
Overall Accuracy	0.529	0.616	0.696	0.693

**Table 8:** Comparison of the performance of ConLoc and Minimalist LR ensemble algorithm with 13 predictors on the Human dataset

	**The best element predictor of ConLoc: Proteome Analyst**	**ConLoc**	**LR ensemble with 13 predictors used in ConLoc**	**LR + minimalist algorithm to select K out of 13 predictors used in ConLoc, K = 3**
Cytosol	0.390	0.414	0.429	0.460
Mitochondrion	0.613	0.628	0.641	0.645
Nucleus	0.463	0.415	0.371	0.392
Secretory	0.754	0.721	0.749	0.758
Overall Accuracy	0.644	0.664	0.689	0.703

To investigate the redundancy among ConLoc’s 13 predictors, we applied our minimalist algorithm to select K out of the 13 predictors and tested them on the Yeast Low-Res dataset and the Human dataset. The results (Tables 7 and 8, column 5) showed that for the Yeast Low-Res dataset, using only 4 predictors can achieve equally good performance as using all the 13 predictors. The most frequent 4 predictors selected by our minimalist algorithm during the 10-fold cross-validation are CELLO, Proteome Analyst, PTS1Prowler, and SherLoc. For the Human dataset, using only 3 predictors can achieve better performance than using all the 13 predictors. The most frequent 3 predictors selected by our minimalist algorithm during the 10-fold cross-validation are Proteome Analyst, PTS1Prowler, and SherLoc.

We also tested PROlocalizer which is an integration algorithm based mainly on binary classifiers. However, the server was able to generate prediction results for only 399 out of 1305 proteins in our Human dataset. The overall prediction accuracy of PROlocalizer on those 399 proteins is 0.81 while the standalone predictor YLoc alone has an overall accuracy 0.84 on the same dataset. We argue that it is difficult to construct a reliable protocol-based ensemble algorithm such as PROlocalizer when the predictions of individual predictors are still not reliable leading to accumulation of errors along its sequential inference steps. Instead, the machine learning based ensemble methods can learn complementary rules among the predictors to function as a “protocol” to determine protein localization.

## Conclusions

Although many protein localization prediction algorithms have been developed, the prediction performance remains low and the features used to predict localizations are still limited. Ensemble algorithms have shown some promise to take advantage of a variety of features by combining individual predictors. However, combining as many as possible individual predictors, which is the most common strategy, has the drawback of high running complexity and low availability as well as risk of performance degradation. The result of our minimalist ensemble algorithm showed that it is possible to significantly reduce the number of individual predictors in a given ensemble algorithm while maintaining comparable performance. It is also observed that the best component algorithm set tends to keep predictors with unique features, which indicates that new features are the key to further improve the prediction accuracy for localization prediction. The success of our minimalist ensemble algorithm based on feature selection and logistic regression showed that supervised ensemble algorithms based on machine learning can effectively capture the complex relationships among individual predictors and achieve better performance than the voting methods.

We found that our ensemble algorithm works best when predictors with unique features are combined. For example, the PPI based NetLoc algorithm can significantly improve the ensemble performance, which is however limited by the fact that many proteins do not have PPI information. It should be also noted that the PPI information and ensemble predictor itself are species specific. So our ensemble predictor trained on human/yeast dataset may not work well for proteins of other species. However, the design methodology of minimalist ensemble predictors can be used to develop predictors tailored to specific organisms or available training datasets.

## Competing interests

The authors declare that they have no competing interests.

## Authors’ contributions

Conceived and designed the experiments: JH;JL. Performed the experiments: JL;AM. Analyzed the data: JL; JH. Contributed reagents/materials/analysis tools: JL;AM. Wrote the paper: JL;JH;RL. All authors read and approved the final manuscript.
